# Management of Hypertension in Pregnancy: Policy Options for Health Care Service Decision-Makers

**DOI:** 10.34172/jcvtr.33933

**Published:** 2026-06-30

**Authors:** Mojgan Mirghafourvand, Leila Doshmangir, Zohreh Alizadeh-Dibazari

**Affiliations:** ^1^Social Determinants of Health Research Center, Tabriz University of Medical sciences, Tabriz, Iran; ^2^Department of Health Policy & Management, Tabriz Health Services Management Research Center, Tabriz University of Medical Sciences, Tabriz, Iran; ^3^Department of Midwifery, TaMS.C., Islamic Azad University, Tabriz, Iran

**Keywords:** Hypertensive disorders, Pregnancy, Policy option, Management, Healthcare policy

## Abstract

**Introduction:**

Hypertensive disorders are a major cause of maternal mortality, and early detection can reduce risks for mothers and infants. Implementing evidence based, context appropriate strategies may improve outcomes. This study aimed to propose policy options to strengthen monitoring and management of hypertension in pregnancy.

**Methods:**

The study consisted of two phases. First, a scoping review synthesized global strategies for improving hypertension management during pregnancy. Searches were conducted in PubMed, Web of Science, Scopus, Cochrane Library, SID, and Google Scholar without time limits, restricted to English and Persian studies. Based on the findings, policy options were developed. In the second phase, 13 experts evaluated these options across five criteria—population benefit, cost/economics, ethics/equity, feasibility, and constituency acceptance—using a five point Likert scale. The top options were further analyzed for advantages, disadvantages, and implementation needs.

**Results:**

From 2,253 identified records, 14 studies informed five policy options: (a) an educational video package on hypertension and preventive self‑care; (b) a maternal mobile application for hypertension management; (c) remote self‑monitoring of blood pressure; (d) a smart multimodal management system; and (e) ambulatory monitoring with Holter devices. Experts ranked the educational video package highest overall and highest for cost/economic, ethics/equity, and feasibility, the mobile application highest for constituency acceptance, and remote blood pressure monitoring highest for population benefit.

**Conclusion:**

Integrating global evidence with expert input yielded three priority policy options: educational video‑based training, a maternal hypertension management app, and remote self‑monitoring of blood pressure. These strategies emphasize empowerment and improved self‑management for pregnant women.

## Introduction

 Pregnancy is a unique physiological process that may nevertheless be accompanied by serious pathological outcomes.^1^ According to maternal health reports, approximately 800 women die every day from preventable causes related to pregnancy and childbirth, with 99% of these deaths occurring in developing countries.^2^ Hypertensive disorders are among the three leading causes of maternal mortality alongside hemorrhage and infection.^3^ Hypertensive disorders of pregnancy affect nearly 10% of all pregnancies and account for 14% of maternal deaths, 15% of perinatal mortality, and 30% of maternal near-miss events worldwide. The International Society for the Study of Hypertension in Pregnancy (ISSHP) classifies hypertensive disorders in pregnancy based on gestational age. Before 20 weeks of gestation, chronic hypertension, white-coat hypertension, and masked hypertension are diagnosed. After 20 weeks, gestational hypertension and preeclampsia are defined by new-onset hypertension accompanied by proteinuria and/or organ dysfunction.^4^ It is important to note that all forms of chronic hypertension regardless of their underlying etiology predispose women to superimposed preeclampsia/eclampsia.^2^ A recent systematic review and meta-analysis reported that the overall prevalence of preeclampsia among Iranian mothers is 5.3%, while eclampsia accounts for 0.1% of live births. Notably, the rate of preeclampsia has increased since 2015, whereas the rate of eclampsia has shown a declining trend over time.^5^

 Early detection of hypertensive disorders in pregnancy can reduce complications for both the mother and the fetus; therefore, regular monitoring of blood pressure and urinary protein levels constitutes an essential component of antenatal care.^6^ The United Kingdom’s Confidential Enquiries into Maternal Deaths (2006–2008) reported that the most common cause of substandard care in deaths related to preeclampsia and eclampsia was the failure to identify and appropriately manage hypertension.^7^ The report for 2009–2014 further indicated that although the proportion of women who died from hypertensive disorders had decreased, improvements in the care provided could have altered the outcomes in 93% of these cases.^8^

 Evidence suggests that complications associated with hypertensive disorders of pregnancy stem from inadequate awareness, negative attitudes toward hypertensive conditions during pregnancy, and insufficient preventive practices among mothers.^9^ One previous study found that 70.7% of pregnant women had poor knowledge regarding hypertensive disorders of pregnancy.^10^ Moreover, research has shown that women with better awareness are more likely to report warning signs promptly and seek timely health care.^11^ Self-care is a conscious, learned, and purposeful activity that individuals undertake to preserve their own lives and safeguard their health and that of their families.^12^ Knowledge of self-care in relation to hypertensive disorders of pregnancy includes understanding skills that help prevent these conditions. Such skills include stress management;^13^ adherence to a diet rich in fruits, vegetables, nuts, whole grains, legumes, olive oil, and fish; restricting the intake of high-fat, salty, and sugary foods—including sugar-sweetened beverages; reducing the consumption of red and processed meats;^14^ engaging in appropriate physical activity during pregnancy;^15^ and maintaining a healthy weight.^16^ Despite this, the study by Salim and Kuriakose reported that 36.4% of pregnant women exhibited poor self-care awareness in this regard.^10^

 Accurate and consistent blood pressure measurement is crucial for the diagnosis and management of hypertensive disorders in pregnancy, which are often asymptomatic. Most deaths associated with these conditions are considered preventable with timely and effective diagnosis, alongside the management of hypertension and its related complications.^8^ Current clinical criteria are typically based on the average of at least two seated blood pressure measurements taken during two separate outpatient visits. Generally, results from home blood pressure monitoring or 24-hour ambulatory monitoring (Holter) tend to be lower than clinical measurements.^17^ There is growing evidence suggesting that home blood pressure monitoring or ambulatory measurements are more strongly correlated with target organ damage compared to measurements taken in physicians’ offices or clinics.^18^ Self-monitoring of blood pressure through home monitoring allows for frequent measurements with minimal disruption to daily activities. This approach is commonly used in managing hypertension in the non-pregnant population within primary and secondary care settings; it is safe, cost-effective, and significantly increases the duration of blood pressure control within the target range.^19^ Given that additional antenatal visits are recommended for monitoring pregnant women with hypertension,^6^ this has led to the exploration of the feasibility of self-monitoring blood pressure in this population.^20^

 In addition to blood pressure measurement, urine protein testing is also routinely performed to screen for the development of preeclampsia. The use of dipsticks to test for urine protein is simple, inexpensive, and rapid, and research has shown that women can perform these tests with accuracy comparable to healthcare professionals.^21^ Consequently, urine testing could be part of a self-care regimen during pregnancy, alongside self-monitoring of blood pressure, to screen for the diagnostic aspects of preeclampsia.

 Given that hypertensive disorders of pregnancy are a major cause of maternal morbidity and mortality around childbirth, examining improvements in the quality of care is of vital importance, particularly following the targets set in 2015 to achieve the Millennium Development Goals. Despite significant resources being allocated to monitor pregnant women with hypertension, both from an individual perspective and from the viewpoint of insurance organizations, it is necessary to adopt an evidence-based approach for the management and control of hypertension in pregnancy. This should be achieved by identifying relevant trials in this field and adapting them to social requirements and local healthcare conditions, based on the perspectives of experts and specialists. Therefore, this scoping review was conducted with the aim of identifying strategies to improve the monitoring and management of hypertension in pregnancy and to provide a policy summary.

## Materials and Methods

###  Study design

 The process of this study involved two stages: (I) a scoping review of global evidence on strategies to improve the monitoring and management of hypertension in pregnancy, and (II) a survey of experts to assess contextual feasibility and assist in formulating policy options. The scoping review was conducted in accordance with the PRISMA-ScR (Preferred Reporting Items for Systematic Reviews and Meta-Analyses extension for Scoping Reviews) guidelines (see Supplementary File 1).

###  Inclusion criteria

 The eligibility criteria for inclusion in the study were defined using the PICOS framework (Study Population, Intervention, Comparison Group, Outcome, and Study Design). Accordingly, studies were included if their study population consisted of pregnant women diagnosed with hypertensive disorders of pregnancy; the intervention studied involved strategies to improve the monitoring and management of hypertension in pregnancy; the comparison group included pregnant women with hypertensive disorders of pregnancy receiving routine care; the outcome studied included maternal and neonatal outcomes (gestational age, duration of maternal hospitalization, mode of delivery, neonatal birth weight, duration of neonatal hospitalization); and the study design included clinical trials and quasi-experimental studies. Review articles, abstracts of studies presented at conferences, and letters to the editor were excluded from this study.

###  Information sources and search strategy

 The databases PubMed, Web of Science, Scopus, Cochrane, SID (Scientific Information Database), and the Google Scholar search engine were systematically searched by two authors (ZA-D & MM) independently until January 30, 2026. There were no time limitations, but the search was restricted to studies published in English and Persian. The following keywords were used:

 (“Pregnancy”[Mesh] OR “Pregnant Women”[Mesh] OR pregnant* OR antenatal OR prenatal OR gestation OR maternal) AND (“Hypertension”[Mesh] OR “Pre-Eclampsia”[Mesh] OR “Blood Pressure”[Mesh] OR “Hypertension, Pregnancy-Induced”[Mesh] OR “high blood pressure” OR preeclampsia OR eclampsia) AND (“Monitoring, Physiologic”[Mesh] OR “Self Care”[Mesh] OR “Telemedicine”[Mesh] OR “Disease Management”[Mesh] OR self-monitoring OR home monitoring OR remote monitoring OR “Blood Pressure Determination”[Mesh] OR management OR strategy OR protocol)

 In addition to the systematic search, a manual search of the references within the articles was also performed. The complete search strategies for all databases are provided in Supplementary File 2.

###  Study selection and data extraction

 Two authors (Z A-D, MM) independently screened the studies for eligibility based on the inclusion and exclusion criteria. Initially, the titles were reviewed, followed by the abstracts. If deemed relevant, the full texts of the studies were then examined. Data extraction was independently performed by the two authors (Z A-D, MM) using a pre-designed form. Any disagreements between the two authors were resolved through consultation with a third author (LD).

###  Development of policy options

 This stage built upon the findings of the scoping review to identify and describe policy options for improving the monitoring and management of hypertension in pregnancy. The objective was to explore how the existing evidence could be interpreted and organized for use in policy planning, considering contextual and social factors. To ensure that contextual and social factors were considered in the interpretation of the review›s findings, 13 experts, including cardiologists, perinatologists, midwives, and reproductive health specialists, were purposefully selected. A structured summary of the interventions identified in the scoping review was provided to them, and they were asked to rate the policy options derived from the scoping review in a questionnaire designed by the researchers. The ratings were based on a 5-point Likert scale (Excellent = 5, Good = 4, Fair = 3, Poor = 2, Very Poor = 1) across five dimensions: population benefit, cost/economics, ethics/equity, feasibility, and constituency acceptance. For each policy option, the average score was calculated for each dimension (possible range 1 to 5). Within each dimension, the policy option that achieved the highest average score was identified as the priority intervention for that dimension. To obtain an overall ranking, the average scores from all five dimensions were summed, giving equal weight to each dimension, which yielded a total possible score ranging from 5 to 25. The three policy options with the highest overall sums were selected as priority policy interventions. Subsequently, each of the priority policy interventions was described, and their reported benefits, limitations, and implementation considerations, in accordance with the included studies, were summarized. Contextual factors such as local health system capacity, available resources, and potential implementation barriers were considered throughout this synthesis.

## Results

###  Identification and selection of studies

 Following a systematic search, complemented by a review of the references of relevant studies, a total of 2253 studies were identified. After the removal of duplicate entries, 1579 studies remained. Of these, 1557 studies were excluded during the title and abstract screening phase due to their lack of relevance to the present study; that is, they either did not meet the criteria for this study or were unrelated to the topic of interest. Subsequently, the full texts of 22 studies were reviewed. This review led to the exclusion of six studies due to inappropriate study design,^22-27^ one study due to an unsuitable intervention,^21^ and one study due to inappropriate participants.^28^ Ultimately, 14 studies ^6,29-41^ were included for the development of policy options (see Figure 1 for the PRISMA flow diagram).

**Figure 1:**
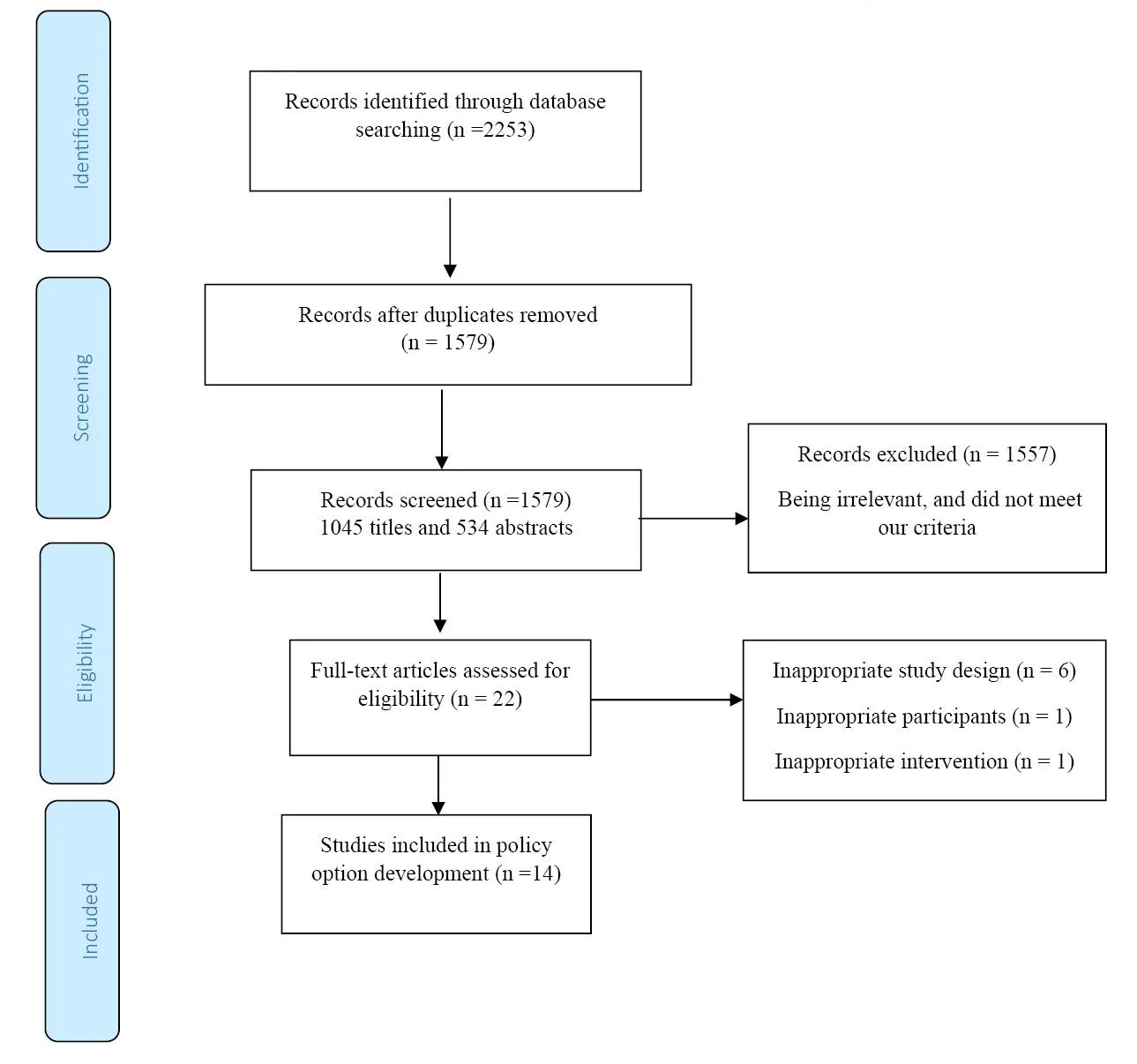
Flow diagram of the systematic literature search

###  Development of policy options

 Based on the conducted scoping review, a total of five policy options were extracted. These options were utilized to aid in contextualizing and clarifying implementation considerations from the perspective of experts. The policy options are as follows:

**A. Development of a video educational package on pregnancy hypertension and self-preventive care: ** In this option, a video educational package, presented in PowerPoint and lecture format, was developed concerning hypertensive disorders in pregnancy and self-preventive care. This package includes classification of hypertensive disorders in pregnancy, predisposing factors, associated signs and symptoms, prevention through weight and stress management, physical activity, nutrition, and regular blood pressure monitoring. It would be distributed individually to pregnant women via domestic and international platforms. A consultant’s contact number would be included with the video to allow individuals to ask questions.^39^ From the experts’ viewpoint, this policy option received an average score of 4.1 for the population benefit dimension, 4.5 for cost/economics dimension, 4.2 for ethics/equity dimension, 4.2 for feasibility dimension, and 4.1 for constituency acceptance dimension, within a obtainable score range of 1 to 5. The calculated overall average score for this policy option was 21.1, out of a potential total score range of 5 to 25.

**B. Development of a mother’s companion mobile application for hypertension care:** This application is designed with five main sections: User profile (including demographic and clinical information of the pregnant mother); Lifestyle (including nutrition and diet education packages, physical activity, and stress management); Disease prevention and control (including a combination of scientific information and practical guidelines for direct management of pre-eclampsia); Application features (including operational tools for implementing the content of the previous sections, such as: introducing specialized treatment centers, medication adherence management, emphasizing the importance of regular medication intake, self-assessment, stress reduction and control, nutrition and diet management, sleep management, contacting physicians, doctor’s appointment reminders, and communication with other patients); and User satisfaction (including periodic surveys, a scoring system, and feedback registration).^35,36^ From the experts’ perspective, this policy option received an average score of 4.3 for population benefit, 4.2 for cost/economics, 4.0 for ethics/equity, 3.8 for feasibility, and 4.3 for constituency acceptance, within a obtainable score range of 1 to 5. The calculated overall average score for this policy option was 20.6, out of a potential total score range of 5 to 25.

**C. Self-monitoring of blood pressure using a remote monitoring system: **In this option, after a medical assessment, pregnant women diagnosed with high blood pressure are identified and registered in an integrated remote monitoring system. An initial electronic health record for the patient is created in the system, including a medical summary, prescribed medications, discharge instructions, and a scheduled self-monitoring plan. This information is then sent to the comprehensive health service center closest to the mother’s residence. A text message is sent to notify the healthcare professional. The patient is trained on how to correctly perform measurements using an automated, pregnancy-validated blood pressure monitor. Patients directly transmit their results to the central system via a mobile application. The system automatically confirms data reception, sends immediate alerts to the patient and care team if dangerous values are detected, and dispatches automated reminders (via SMS or app notification) if measurements are not sent on time. All data are displayed in visual charts within the patient’s electronic health record and are accessible to the physician during follow-up visits. Based on the self-monitoring data and the system’s intelligence, the care professional can follow up with the patient via phone or video call, summon the patient for an in-person visit to the health center or hospital if necessary, and update medications or instructions. These changes are also logged in the system and communicated to the patient.^31,32,37^ From the experts’ perspective, this policy option received an average score of 4.5 for population benefit, 3.3 for cost/economics, 3.8 for ethics/equity, 3.2 for feasibility, and 4.1 for constituency acceptance, within a obtainable score range of 1 to 5. The calculated overall average score for this policy option was 18.8, out of a potential total score range of 5 to 25.

**D. Intelligent multimodal management system for gestational hypertension:** In this option, an intelligent multimodal application is provided to both the patient and the treatment team. A Bluetooth-enabled blood pressure monitor automatically transmits measurements to the application and subsequently to the central electronic medical records system. Urine protein testing kits (e.g., urine dipsticks) are provided to the patient. The patient enters the test results into the application. Patients can also log symptoms such as headache, blurred vision, chest pain, etc., within the application. A clinical algorithm, designed based on standard protocols, analyzes blood pressure and proteinuria data. This algorithm provides suggestions for adjusting antihypertensive medication dosages. These suggestions are forwarded to a specialist physician or midwife for final approval, and upon confirmation, the new instructions are automatically communicated to the patient. The application includes an educational section with written information, short videos, and infographics about pre-eclampsia, the importance of medication adherence, warning signs, and a healthy lifestyle. The central system monitors the data 24 hours a day. In cases of critical data reception (very high blood pressure, severe proteinuria, reported warning symptoms), an immediate alert is automatically sent to the care team (physician, midwife, healthcare professional), along with an immediate feedback message and initial instructions to the patient.^6,41^ From the experts’ perspective, this policy option received an average score of 4.1 for population benefit, 2.9 for cost/economics, 3.6 for ethics/equity, 2.8 for feasibility, and 4.0 for constituency acceptance, within a obtainable score range of 1 to 5. The calculated overall average score for this policy option was 17.4, out of a potential total score range of 5 to 25.

**E. Ambulatory blood pressure monitoring using Holter monitoring devices:** In this option, a Holter monitoring device, which includes a small and specialized blood pressure monitor, is first programmed and configured by a computer and then attached to the patient. This tool automatically measures the patient’s blood pressure at specified intervals – every half hour during the day and every two hours at night for a 24-hour period – while the patient is engaged in their daily activities. During prenatal visits, the blood pressure control level is reviewed by examining the device’s data.^30,40^ From the experts’ perspective, this policy option received an average score of 3.8 for population benefit, 2.8 for cost/economics, 3.6 for ethics/equity, 2.6 for feasibility, and 4.1 for constituency acceptance, within an obtainable score range of 1 to 5. The calculated overall average score for this policy option was 16.9, out of a potential total score range of 5 to 25.

 The advantages, disadvantages, and implementation considerations of the policy options are presented in Table 1.

**Table 1:** Advantages, disadvantages, and implementation considerations of policy options for improving monitoring and management of hypertension in pregnancy.

**Policy Options**	**Advantages**	**Disadvantages**	**Implementation Considerations**
Development of a video educational package on pregnancy hypertension and self-preventive care	• Increased knowledge for pregnant women regarding types of gestational hypertension, signs and symptoms, risk factors, complications, and prevention ^42^. • Enhanced understanding of self-care in gestational hypertension ^39^. • Earlier diagnosis and management of gestational hypertension, reducing complications and mortality by improving pregnant women’s knowledge ^39^. • Usefulness of video training due to information transfer in a short time, pre-defined content, ability to store more information, information consistency, avoiding confusion in training, and possibility of adding new information to previous content ^43^.	• Ineffectiveness of educational content due to differing learning needs of each woman based on age, socioeconomic status, previous experiences, and expectations ^44^. • Inducing feelings of threat and uncertainty about health status in pregnant women ^45^.	• Educational content must align with women’s needs ^46^. • Educational content should have a woman- and family-centered framework including participatory activities for the needs of women and their families during pregnancy, childbirth, and postpartum ^47^.
Development of a mother’s companion mobile application for hypertension care	•Provision of reliable and accurate information to users anytime and anywhere ^48^. •Improvement of self-management status in pregnant women ^49^. • Effective communication with healthcare providers ^49^. • Improvement of lifestyle in pregnant women with hypertension, considering basic psychological needs such as motivation and self-determination ^35^. • Reduction in frequent in-person visits to doctors’ offices, decreased workload for physicians, time-saving for them, and reduced risk of infectious diseases ^36^.	• Increased likelihood of cyberchondria - excessive and anxiety-provoking online medical information seeking ^50^. • Creation and exacerbation of a vicious cycle of frequent searches, increased worry, and reinforcement of negativity ^51^. • Need for a smartphone.	• In countries like Iran, due to widespread smartphone access, mobile-based e-health can be used for personalized, sustainable, and adaptive primary and secondary prevention interventions to improve antenatal care and maternal and fetal health outcomes ^35^. • Emphasis should be placed on the necessity for healthcare providers to address internet hypochondria and associated anxiety in pregnant women through comprehensive psychological support, reliable guidance, and targeted educational interventions ^52^.
Self-monitoring of blood pressure using a remote monitoring system	• Saving time, reducing stress, travel costs, and time off work ^22^. • Reduced burden of multiple clinic visits for pregnant women and their healthcare teams ^53^. • Motivating pregnant women and keeping them active in self-care ^37^. • Effective participation and good communication between patient and specialists ^24^. • Assistance in shared decision-making ^24^. • No higher cost than routine care ^41^.	• Greater trust from specialists in clinic-based blood pressure monitoring compared to self-monitoring ^54^. • Patient’s attempt to achieve normal blood pressure values by frequent measurement and selective or delayed reporting if high values are measured ^41^. • Lower blood pressure readings in home measurement compared to clinic measurement as a justification for not starting antihypertensive medication ^41^. • Uncertainty about how to incorporate self-monitoring blood pressure results into the clinical decision-making process ^31^.	• Adding blood pressure self-monitoring to routine antenatal care appears to be both safe and acceptable ^55^. • Consideration of the costs of implementing a remote monitoring system at a national level, especially given the very large target population, is crucial for policymakers and planners ^31^.
Intelligent multimodal management system for gestational hypertension	• Provides real-time, automated transmission of blood pressure and proteinuria data to the central system, reducing recall bias and manual entry errors ^6,41^. • Clinical algorithm offers suggestions for antihypertensive dose adjustments, which may accelerate clinical decision-making ^6^. • Immediate alerts for critical values (very high BP, severe proteinuria, warning symptoms) enable rapid response by the care team ^41^. • Educational section (written, video, infographics) reinforces self-management and warning sign recognition ^6^.	• High implementation costs due to Bluetooth-enabled monitors, urine dipstick kits, and algorithm development/maintenance ^31,53^. • Low feasibility in low-resource settings because of dependence on continuous smartphone/Internet connectivity and a functioning electronic medical record system ^38,49^. • Requires specialist oversight (physician or midwife) to approve algorithm-generated suggestions, which may not be available in rural areas ^24,32^. • Potential for alert fatigue among clinicians if many patients generate frequent non-urgent notifications ^22^.	• Reliable electronic health record system capable of receiving and displaying multimodal data (BP, proteinuria, symptoms) ^6,41^. • Validated Bluetooth-enabled BP monitors and affordable urine dipstick kits for home use ^21,37^. • Defined clinical protocols for algorithm approval, escalation of alerts, and medicolegal responsibility for algorithm-guided dosing ^32^. • Initial training for both pregnant women (using devices, logging symptoms) and healthcare providers (interpreting automated suggestions) ^24^. • Contingency plan for patients without smartphones (e.g., community health worker data entry or SMS-based alternatives) ^29,49^.
Ambulatory blood pressure monitoring using Holter monitoring devices	• Provides 24-hour blood pressure profile during normal daily activities, capturing nocturnal and diurnal variations ^30^. • Helps differentiate white-coat hypertension from true hypertension, potentially avoiding unnecessary interventions ^40^. • Measurements are automated and objective, not subject to patient recording errors ^30^. • Can assess adequacy of antihypertensive treatment over a full day–night cycle ^30^.	• Requires patients to return to a clinic or hospital to have the device attached and later removed, which is burdensome for pregnant women, especially in remote areas ^40^. • Holter devices are expensive, require programming, and are not suitable for continuous long-term monitoring (typically only 24 hours) ^30^. • Does not provide real-time data or alerts; results are reviewed retrospectively, delaying response to acute hypertension ^41^. • Discomfort or sleep disturbance from repeated cuff inflations may reduce compliance ^30^.	• Availability of validated ambulatory BP monitors (Holter devices) with appropriate cuff sizes for pregnant women ^30,40^. • Trained personnel to program, attach, and download data from devices ^30^. • A scheduled return visit for device removal and data interpretation, which adds to clinic workload and patient travel ^40^. • Clear criteria for interpreting ambulatory BP values in pregnancy (currently less standardised than in non-pregnant adults) ^17,54^. • Backup plan for device malfunction or patient intolerance (e.g., home BP monitoring as an alternative) ^37^.

## Discussion

 In the present study, based on a scoping review, 14 studies related to strategies for improving the monitoring and management of hypertension in pregnancy were identified, leading to the identification of five policy options. Among these policy options, “Development of a video educational package on pregnancy hypertension and self-preventive care” received the highest average scores in the cost/economics dimension, the ethics/equity dimension, and the feasibility dimension, as well as the highest overall average score. “Development of a mother’s companion mobile application for hypertension care” achieved the highest average score in the constituency acceptance dimension, and “Self-monitoring of blood pressure using a remote monitoring system” obtained the highest average score in the community benefit dimension.

 The policy option “Development of a video educational package on pregnancy hypertension and self-preventive care” ranked highest among other policy options in terms of average scores for the cost/economics dimension, the ethics/equity dimension, and the feasibility dimension, as well as the highest overall average score. In line with this, various studies have shown that women with good awareness of hypertension during pregnancy are more likely to report symptoms promptly and seek medical care.^11,56^ In fact, self-care is a conscious, learned, and purposeful action that an individual undertakes to maintain their life and the health of themselves and their family.^12^ This is despite the fact that a study by Sarabi et al, conducted on pregnant women attending health centers in Dezful, Iran, revealed that more than half of the study participants had no prior knowledge of hypertension in pregnancy, a figure significantly higher than in similar studies.^39^ Given that hypertensive disorders in pregnancy are a major contributor to maternal complications and mortality, education in this area must be prioritized in health programs to achieve the World Health Organization’s goal of reducing maternal mortality by 2030.^57^ Meanwhile, rapid advancements in digital health technologies offer transformative opportunities for managing chronic diseases.^58^ Telemedicine, E-Health, and mobile health (M-Health) enable patients to actively monitor and improve their health while enhancing the quality of healthcare services. These innovative approaches are particularly important for managing chronic conditions like hypertension, which require continuous self-care and regular interactions between patients and their healthcare teams.^59^ As pregnant women may face certain infrastructural challenges in accessing healthcare facilities, M-Health programs and wearable devices can eliminate the need for physical referrals. They provide pregnant women with hypertension with real-time feedback, personalized education, and self-monitoring tools to encourage the adoption of healthier behaviors.^49^ The policy option “Development of a mother’s companion mobile application for hypertension care” aligns with this direction, receiving the highest average score in the constituency acceptance dimension among the policy options in this study. Molaei et al. designed, developed, and evaluated a mobile-based application to facilitate self-care in pregnant women with pre-eclampsia during the COVID-19 pandemic. The results from this study on pregnant women in Kerman, Iran, indicated that the application helped reduce stress in pregnant women, increased users’ knowledge, improved their attitudes towards COVID-19 and pre-eclampsia, enhanced their self-care and self-management processes, and ultimately led to a less challenging pregnancy and delivery. The application also reduced the frequency of in-person visits to physicians, decreased physicians’ workload, and lowered the potential risk of COVID-19 transmission for both patients and physicians.^36^

 The results of this study indicated that the third policy option, “Self-monitoring of blood pressure using a remote monitoring system,” is also in line with telemedicine and the promotion of self-monitoring and self-care for pregnant women. This policy option received the highest average score in the community benefit dimension. Clinical trial results conducted in England demonstrated that self-monitoring of blood pressure using a remote monitoring system did not lead to earlier detection of hypertension or better control of it. However, more than half of the participants diagnosed with hypertension had elevated blood pressure during home measurements, and the lack of impact was partly attributed to uncertainty regarding the integration of self-monitoring blood pressure results into the clinical decision-making process. Reassuringly, individuals who performed self-monitoring did not experience any additional harm, unexpected outcomes, or adverse effects on their health-related quality of life.^6,32^ In another trial conducted by Pealing et al. on pregnant women with gestational hypertension and chronic hypertension across four centers in England, it was shown that blood pressure readings recorded at the clinic and those from self-monitoring were similar for both groups. These researchers concluded that self-monitoring of blood pressure is feasible and tolerable for managing hypertension during pregnancy when combined with clinical supervision.^37^

 When translating these policy options into real-world practice, several barriers and facilitators must be considered. Digital literacy varies considerably among pregnant women, and while the educational video package can be distributed via offline media (e.g., USB drives or clinic-based viewing) and therefore remains accessible even to those with limited digital skills,^39^ the mobile application and remote monitoring options presuppose basic proficiency with smartphones and health apps.^35,36,48^ Access to smartphones, though increasingly widespread, is not universal, particularly among lower-income or rural populations;^38^ this poses a risk of exacerbating health inequities if digital health solutions are implemented without providing alternative means of participation, such as community health worker-assisted data entry or simplified text message-based systems.^29,49^ In low-resource or remote settings, the intelligent multimodal system and Holter monitoring are likely to be least feasible due to equipment costs, maintenance requirements, and dependence on stable connectivity,^31,53^ whereas the video educational package and even paper-based complements to the mobile app can be designed for offline use. Health-system integration represents another critical challenge: remote blood pressure monitoring and the intelligent system require clear protocols for transmitting self-measured data into electronic health records, defining who is responsible for reviewing alerts, and ensuring that additional clinician workload is recognised and compensated.^6,22,24^ Without such integration, even effective technologies may be underutilized or lead to fragmented care. Facilitators that can overcome these barriers include phased implementation starting with higher-risk or urban populations, involving end-users (both pregnant women and healthcare providers) in co-designing interventions,^23^ and embedding digital health tools within existing maternal and child health programmes rather than introducing them as standalone additions.^6,32^ Furthermore, addressing internet-related health anxiety (cyberchondria) through reliable guidance and psychological support is essential when using app-based interventions.^50-52^ Ultimately, no single policy option will fit all settings; a tailored approach that matches the local level of digital infrastructure, health system capacity, and population literacy is essential for equitable and sustainable improvement in hypertension management during pregnancy. It appears that a large-scale randomized controlled trial assessing the efficacy and cost-effectiveness of self-monitoring for improving blood pressure control in pregnancies with hypertension is warranted.

## Strengths and limitations

 This scoping review synthesizes findings from 14 clinical trials, providing evidence on effective interventions for improving the monitoring and management of hypertension in pregnancy. The use of a structured scoping review methodology, combined with the insights from 13 experts in cardiology, perinatology, midwifery, and reproductive health, enhances the interpretation of contextual factors and supports the identification of interventions applicable across diverse healthcare settings. However, this study only examined texts published in Persian and English. Consequently, texts in other languages and unpublished data regarding strategies for improving the monitoring and management of hypertension in pregnancy were not considered. Furthermore, some of the included texts involved interventions conducted in countries other than Iran. Therefore, it is recommended that future research investigate the practicality and effectiveness of the policy options proposed in the present study and prioritize them based on their efficiency and efficacy.

## Conclusion

 Monitoring and managing hypertension in pregnancy necessitates interventions that are practical, flexible, and tailored to specific circumstances. This study identified three key policy options: (a) development of a video educational package on pregnancy hypertension and self-preventive care, (b) development of a mother’s companion mobile application for hypertension care, and (c) self-monitoring of blood pressure using a remote monitoring system. These strategies aim to empower women and enhance their self-management, self-care, and self-monitoring capabilities. Healthcare systems can contribute to improving maternal health and reducing complications from high-risk pregnancies by creating an environment that facilitates women’s effective participation in self-care and fosters strong connections between pregnant women and community health workers. This, in turn, can reduce the workload of health centers, leading to long-term social and economic benefits. The findings of this study underscore the need for collaboration among policymakers, healthcare providers, and service recipients to adapt and integrate evidence-based and culturally sensitive approaches to support women experiencing high-risk pregnancies.

## Competing Interests

 No conflicts of interest to declare.

## Ethical Approval

 The data, models, and methods used in this study are not proprietary and no human participants were involved in this study. However, the study adheres to the ethical conduct policy outlined by the International Committee of Medical Journal Editors (ICMJE) (http://www.icmje.org/). This study was approved by the Ethics Committee of Tabriz University of Medical Sciences, Tabriz, Iran(Approval No: IR.TBZMED.REC.1401.936).

## Supplementary Files

Supplementary File 1 contains PRISMA-ScR guidelines (unchanged, but now referenced in manuscript).

Supplementary File 2 contains search strategy For systematic literature review for all databases.
