# Supplementary material for: Management of Hypertension in Pregnancy: Policy Options for Health Care Service Decision-Makers
Source: J Cardiovasc Thorac Res. 2026 Jun 30;18(2):123–32. doi: 10.34172/jcvtr.33933 (PMC13677606; doi:10.34172/jcvtr.33933)
Supplement: Supplementary file 2 [file jcvtr-18-123-s002.pdf]

J Cardiovasc Thorac Res 2026;18(2):123-S2
doi:10.34172/jcvtr.33933
https://jcvtr.tbzmed.ac.ir

PubMed Search Strategy

((((((((Pregnancy[Mesh]) OR ("Pregnant Women"[Mesh]) OR (pregnant*[Text Word]) OR (antenatal[Text Word]) OR (prenatal[Text Word]) OR (gestation[Text Word]) OR (maternal[Text Word])) AND ((((((((Hypertension[Mesh]) OR ("Pre-Eclampsia"[Mesh]) OR ("Blood Pressure"[Mesh]) OR ("Hypertension, Pregnancy-Induced"[Mesh]) OR ("high blood pressure" [Text Word]) OR (preeclampsia[Text Word]) OR (eclampsia[Text Word])) AND ((((((((((("Monitoring, Physiologic"[Mesh]) OR ("Self Care"[Mesh]) OR ("Telemedicine"[Mesh]) OR ("Disease Management"[Mesh]) OR ("self-monitoring" [Text Word]) OR ("home monitoring" [Text Word]) OR ("remote monitoring" [Text Word]) OR ("Blood Pressure Determination"[Mesh]) OR (management [Text Word]) OR (strategy[Text Word]) OR (protocol[Text Word]))

No time limits; English or Persian.

Web of Science (WoS) Search Strategy

1: ((((((((((TS=("Fear of childbirth")) OR TS=(Tokophobia)) OR TS=("Childbirth fear")) OR TS=("Birth fear")) OR TS=("Labor anxiety")) OR TS=("Delivery anxiety")) OR TS=("Prenatal anxiety ")) OR TS=("Childbirth-related anxiety")) OR TS=("Fear of labor")) OR TS=("Fear of delivery")) OR TS=("Obstetrical phobia")

1: ((((((TS=(Pregnancy)) OR TS= ("Pregnant Women")) OR TS=(pregnant)) OR TS=(antenatal)) OR TS=(prenatal)) OR TS=(gestation)) OR TS=(maternal)

2: ((((((TS=(Hypertension)) OR TS= ("Pre-Eclampsia")) OR TS= ("Blood Pressure")) OR TS= ("Hypertension, Pregnancy-Induced")) OR TS= ("high blood pressure")) OR TS=(preeclampsia)) OR TS= (eclampsia)

3: ((((((((((TS= ("Monitoring, Physiologic")) OR TS= ("Self Care")) OR TS= ("Telemedicine")) OR TS= ("Disease Management")) OR TS= ("self-monitoring")) OR TS= ("home monitoring")) OR TS= ("remote monitoring")) OR TS=("Blood Pressure Determination")) OR TS=(management)) OR TS= (strategy)) OR TS= (protocol)

4: #1 AND #2 AND #3

Scopus Search Strategy

TITLE-ABS-KEY(Pregnancy OR "Pregnant Women" OR pregnant* OR antenatal OR)prenatal OR gestation OR maternal) AND TITLE-ABS-KEY (Hypertension OR "Pre-Eclampsia" OR "Blood Pressure" OR "Hypertension, Pregnancy-Induced" OR "high blood pressure" OR preeclampsia OR eclampsia) AND TITLE-ABS-KEY("Monitoring, Physiologic" OR "Self Care" OR "Telemedicine" OR "Disease Management" OR "self-monitoring" OR "home monitoring" OR "remote monitoring" OR "Blood Pressure Determination" OR management OR strategy OR protocol)

Cochrane Library Search Strategy

1. (Pregnancy OR "Pregnant Women" OR pregnant* OR antenatal OR)prenatal OR gestation OR maternal). ti,kw,ab.

2. (Hypertension OR "Pre-Eclampsia" OR "Blood Pressure" OR "Hypertension, Pregnancy-Induced" OR "high blood pressure" OR preeclampsia OR eclampsia). ti,kw,ab.

3. (“Monitoring, Physiologic” OR “Self Care” OR “Telemedicine” OR “Disease Management” OR “self-monitoring” OR “home monitoring” OR “remote monitoring” OR “Blood Pressure Determination” OR management OR strategy OR protocol). ti,kw,ab.

4. 1 and 2 and 3
